# Four Versions of Transtheoretical Stances, and the Bernese View

**DOI:** 10.32872/cpe.12453

**Published:** 2024-04-26

**Authors:** Franz Caspar, Thomas Berger

**Affiliations:** 1 Department of Clinical Psychology and Psychotherapy, University of Bern, Bern, Switzerland; Department of Psychology, University of Trier, Trier, Germany

**Keywords:** transtheoretical, psychotherapy integration, multiple constraint satisfaction, individualization, supervision

## Abstract

A brief characterization of transtheoretical stances to which existing approaches can be allocated is followed by a description of the "Bernese view", that is, what Klaus Grawe and his colleagues, including the authors of this article have developed: the origins, a model of the multiple constraint satisfaction construction of therapist action, a discussion of psychotherapy integration, the crucial role of supervisors in an integrative multiple constraint satisfaction approach, and a discussion of when and how trainees should be introduced to a transtheoretical stance.

## Highlights

- Various understandings of transtheoretical exist.
- Two differences are particularly important: The degree to which theories are integrated, as opposed to conveyed/used as separate approaches, and the striving for "the" right approach vs. emphasizing that we should rather expect and further a never-ending process.
- A concept of satisfying multiple constraints in the moment to moment construction of therapist action is more realistic than adapting standardized procedures.
- Using multiple theoretical concepts is advantageous in the endeavour of offering an optimally fitting psychotherapy.

## Your Personal Preference

Let's assume *you* have (or a person you really care about has) a major psychological problem. Your problem may fit into a diagnostic category (such as major depression, or one or the other kind of anxiety disorder) or not, in any case, in the individualized assessment of problems and treatment goals (Goal Attainment Scaling; Kiresuk et al., 2014), the reduction of depression (or anxiety) would appear as secondary or third treatment goal only. More concerned you are about the fact that you have severe conflicts with your adolescent children, or being treated badly at work. The work-related problems you see as a consequence of mobbing.

Let's also assume that as a psychotherapy-interested person, you are informed about the approximative contribution to outcome of a specific approach, the therapist, and the relationship. Still, you are also aware that these are averages, while for the individual patient, a detail may make all the difference. Let's assume that you are also familiar with research showing that professional top performers in general, are contextually oriented. That is, they consider a wealth of circumstances before and while they act (Caspar, 2017), and this is in line with your own professional experience in a different field. What kind of treatment and what kind of therapist would you look for? Let's leave this question open at the moment.

## Four Transtheoretical Stances

While many variations are possible, we see four major ways in which a therapist or training program can be transtheoretical:

1. Learn several approaches to psychotherapy (e.g., psychodynamic and behavioral) in parallel or sequentially and practice somehow based on a limited number of pure approaches.
2. Strive towards the one coherent approach that integrates several existing approaches to psychotherapy.
3. Use elements of various origin in an integrative way while maintaining the view that various approaches should maintain differences, as this is a precondition for an ongoing dialectical process in which many therapists and researchers actively participate.
4. Start with a particular, ideally already integrative, approach, then strive for continuous development, integrating further conceptual and practical elements not only from existing approaches to psychotherapy, but also basic science. Not only integrating what appears useful but also dealing with contradictory concepts and evidence.

The first version, while open beyond one single approach, sticks to a view of the world of psychotherapy as divided into approaches. The stance is practiced in institutions seeing themselves as transtheoretical as not one single approach is taught, but if the exchange between these approaches is limited, one might say, it's "transtheoretical light". To the defense of this version, one might say that such a version is based on history, and, after all, somehow complexity needs to be reduced. Critically one might say that in this version, the goal of *integrating* the approaches is not dominating, and the individual therapist is, by and large, left alone with the task of combining conceptual and interventive elements in practice.

The second version is attractive for institutes or individuals intending to provide and "sell" THE integrative approach in general, or use integration in favor of an approach with more limited claims of validity (such as CBASP for chronic depression; McCullough, 2000). The risk of this variation is that unlike versions three and four, there is a considerable chance of petrification and a development to just another approach defending its superiority.

The third version is well represented in the Society for the Exploration of Psychotherapy Integration (SEPI). As a SEPI steering committee member, one of us (FC) has participated in many discussions about whether SEPI should switch to tempting version two and certify therapists as SEPI-proved integrative therapists aiming at the gaining of attractiveness. But always those have won who defended the dialectical version three, which is also signaled by the unwieldy name component "exploration of …". As Wolfe (2000) states: “… only a minority of SEPI members believes it is even possible to develop an integrative psychotherapy theory. Even if it were possible, such a theory would not be a great idea, some argue, because it would have a chilling effect on therapeutic creativity” (p. 234). Most colleagues would, while acknowledging the importance of guidance coming from theoretical concepts, agree that none of the existing theories satisfies all needs and preferences (Walder, 1993), and that maintaining a variety of approaches may be the best and maybe only antidote against the loss of diversity which may prove useful when it comes to new challenges – analogously to biological diversity and gene pools.

While to some extent compatible with this third version, the fourth version, corresponding to the Bernese view, based on Grawe's *General Psychotherapy*, seems to incorporate most advantages, and will therefore get more space here.

## The Origins of the Bernese View

In the mid 1970s, Grawe and colleagues, working at the Hamburg Eppendorf University Psychiatry Hospital, were confronted with patients with mixed diagnoses who were treated in behavioral group therapies. From a technical point of view, therapists did everything right, but some patients did not really engage in therapy and brought various difficulties into the therapeutic relationship. While it was not common at that time to use personality disorder diagnoses, nowadays, many of them would be characterized by such diagnoses. Grawe developed a form of case formulation that focused more on the motivational background of problem behavior and its instrumental function. This overriding of some of the limits of behaviorism allowed the development of individualized "complementary" strategies in the therapeutic relationship. Of influence on his thinking was also the fact that at that time, research on Client Centered Therapy (in which he also had a partial training) showed equally good results as Behavior Therapy, even with anxiety disorders, which many considered to be a domain of Behavior Therapy (Grawe, 1976).

In the early 1980s, Caspar developed Grawe's "Vertical Behavior Analysis" further with particular respect to the role of emotions and the analysis of the patients' problems. These additions led to a replacement of the name "Vertical Behavior Analysis" with "Plan Analysis" (Caspar, 2018b, 2022). In Plan Analysis an instrumental perspective is taken: For conscious and non-conscious, interpersonal and intrapsychic behaviors it is asked what purpose or motives they serve. Plans are the basic unit of analysis: they consist of a motivational component and means serving this motive. The two-dimensional Plan structure represents the whole of inferred strategies of a person, ordered in a hypothetical instrumental hierarchy with concrete behaviors on the bottom and general needs on top. Plan Analysis case formulations serve two major purposes: To understand the functioning of patients in the therapy relationship, and the development and maintenance of psychological problems. These can be a consequence of instrumental strategies (e.g., a depression developing when a person avoids leaving home for two years to avoid agoraphobic anxieties), or they can be means serving the solution of problems (e.g. a depression serving the hypothetical purpose of avoiding the conflict in a difficult pro/con decision related to a potential coming out by a homosexual person in a homophobic environment). Plan Analysis is neutral as far as schools of therapy are concerned, and various approaches to therapy can guide the hypothesis generation: instrumental conditioning: behavior therapy; testing the therapist with challenging behavior in the relationship: psychodynamic control-mastery approach; avoidance of threating emotions by transformation into emotions that are less threatening on a short by maladaptive on a long range: Emotion Focused Therapy; etc.).

In a major RCT, a Plan Analysis based form of Broad Spectrum Behavior Therapy has been compared to a form of Broad Spectrum Behavior Therapy based on traditional behavior therapy case formulations (Lazarus, 1971), and classical Client Centered Therapy without any explicit form of case formulation (Grawe, Caspar, & Ambühl, 1990). Plan Analysis based therapies fared better in some outcome criteria, but not pervasively, while the process was stunningly more favorable from patient, therapist and observer perspectives.

An important feature of this study was that the three RCT conditions were not defined in a narrow algorithmic but in a rather heuristic way by prescribing three different approaches in terms of case conceptualization while leaving the concrete procedure open as long as it was based on the respective individual case conceptualization. Of course, it was considered necessary to know how therapists proceeded concretely, but this was described retrospectively based on video analyses. These showed by far the most technical and conceptual richness in the Plan Analysis condition, including the reference to psychodynamic and gestalt therapeutic elements. The justification for and advantages of such a heuristic, integration-friendly form of RCTs is described in more detail in Caspar (2018a), for a recent study related to the assimilative integration of Emotion Focused elements see Caspar et al. (2023).

Grawe (1998, 2004) termed the classical therapy orientations *first-generation approaches*. Typical for them is that usually, charismatic founders formulate a coherent approach incorporating theoretical and practical concepts. Typically, they reinforce and defend their approach against competing approaches by (over-) emphasizing its advantages and, more often than not, ignoring or suppressing information incompatible with their assumptions, and fostering group thinking: "we, the good and smart ones, own the best concepts and our patients are blessed that they can profit from this. The others are ignorant, ineffective, not thorough enough, even unethical, etc." *Second-generation approaches*, in contrast, are open to dealing with concepts and findings challenging their existing views, and continually strive to deal with evidence, integrating useful parts not only of alternative existing approaches to psychotherapy, but also of insights from basic and applied science. The ideal of reaching a point of saturation is asymptotic, that is, it is never reached, because approaches of psychotherapy ever evolve further, and so does science. This stance has been denominated *General Psychotherapy*. It is not yet another approach with content and techniques, let alone a transdiagnostic model striving to correspond to the second type (see above), but a model for a continuous process with which a holding on to a particular state in the development would be incompatible. The explicit reference to other concepts and approaches, new and old ones, in a continuous process of change, is a bastion for scientific honesty and appreciation for concepts of others, which is a value considered crucial for further development.

Grawe has compared first generation approaches to "Konfessionen" (German for religious denominations) and entitled a book "Von der Konfession zur Profession" (from religion to profession; Grawe, Donati, & Bernauer, 1998), that is, characterizing the behavior of founders and followers of first generation approaches as unprofessional. The direction of development should, of course, be from confession to profession. It is hard to avoid seeing many developments within the "third wave of Behavior Therapy" going the other way: "From profession to confession" by uncritically following (more or less charismatic) leaders and believers, who don't justify their actions based on a comprehensive individual case formulation but rather do what their approach suggests: "I'm doing this, because I'm an XY-therapist."

For the Bernese approach, a clear emphasis was all along on individual case conceptualizations as a basis for custom-tailoring the therapy to the patient and the concrete situation on the level of relationship as well as working on the problems. Plan Analysis captures the recurrent patterns while being open to systematic variations across situations. The therapist adapts to stable patient characteristics as well as to characteristics and particularities in the moment. Grawe (1988) has entitled one article, arguing for a rather heuristic than algorithmic, process-oriented understanding of therapy with "Der Weg entsteht beim Gehen" (the path develops as one walks it). Such a view of therapy has been grounded for him and FC in training in humanistic therapies.

This corresponds to the idea of "contextual" acting, which is typical for top performers in various professions (Caspar, 2017; see below) and is also seen as a precondition for "responsiveness" in psychotherapy (Stiles, 2021). Responsiveness (overlapping with the terms personalization and precision psychotherapy) is across orientations one of the most topical issues in the current psychotherapy discussion when asking how we can further improve psychotherapy (see, e.g., programs of international psychotherapy conferences).

## A Model of Creative Multiple Constraint Satisfaction

**Figure 1:**
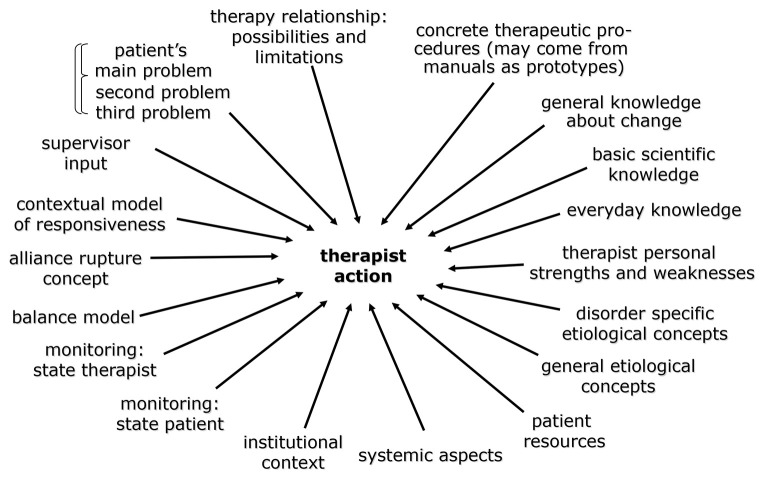
Creative Construction Model *Note.* Model of parallel multiple constraint satisfaction in the construction of therapist action. The brackets at the problems indicate that they are typically interconnected. The list of aspects to be considered is not exclusive (adapted from Caspar, 2000, 2022).

A therapist should be contextual, that is, able to include as many relevant properties of the patient and the situation as possible. A model for this we call “multiple constraint satisfaction model of constructing therapeutic action anew” or “Creative Construction Model” (Figure 1)

The model may appear pretty complex, yet it is assumed to represent what good, experienced therapists include in their construction process. Three assumptions are important:

1. Not all aspects are equally relevant in each case and situation: a patient with a straightforward agoraphobia without instrumental function, easy in the therapeutic relationship, requires less multiple constraint satisfaction than average.
2. Such a multiple constraint satisfaction process is, once a therapist has practiced it several times in a conscious, step-by-step manner, relatively intuitive. The processes become fast and thus workable.
3. Novices can't be expected to master the complexity of all potentially relevant aspects from the outset. It is temporally the supervisor's task to make sure that no crucial aspect gets neglected (see below).

## The Development of Expertise

Therapist information processing (hypothesis generation, decision making, etc.) and the development of expertise in such processing has been a main working area of both authors of this article, and Cognitive Science is seen as providing models and language suitable for neutral transtheoretical reflection and discussion. The question of when and how in their development psychotherapists can deal with how much complexity is crucial when advocating the described creative construction model vis a vis psychotherapy trainees. For an answer, it is obvious to look into the literature on the development of professional expertise. There are several phase models for the professional development of therapists. A non-clinical model of high relevance stems from Dreyfus and Dreyfus (1986). According to them in an initial phase, professionals stick to clear, simple when-then rules, and to relatively simple models. This simplicity is appropriate for their beginning level of development, but the results are at the same time considered to be suboptimal. The experience of limitations with individual tasks/cases is seen as the driving force behind a process of enlarging perspectives as well as concrete procedures. According to the Dreyfus and Dreyfus model, the subjective confidence first *decreases* instead of *increasing* as the therapist gains experience. This is due to the awareness that multiple perspectives are possible, and that the responsibility of the therapist is not only to use rules properly, but also to decide on the right perspective or combination of concepts. In the later stages of professional experience psychotherapists develop an ease and efficiency in the form of a good combination of rationally and intuitively knowing what is right, a process which is expected to take about 10 years (Caspar, 2017; Ericsson, Krampe, & Tesch-Römer, 1993), and leads to the ability to and practice of including a big number of aspects, or contextuality.

## Psychotherapy Integration

The advantage of psychotherapy integration is obvious from the perspective of a model of continuously constructing therapist action anew:

Widening the perspective and the tool-box, and breaking free from the limitations of one single approach is supposed to increase the a priori chance of finding the optimal view and procedure in the sense of maximal desired main and positive side effects, accompanied by minimal negative side effects. All possible procedures have negative side effects, sometimes relatively harmless, but often more severe. The more flexibility, the higher the chance to succeed with an optimal main/side effects balance – unless the therapist fails in mastering the complexity. (Caspar, 2010, pp. 18-19)

This chance for optimizing psychotherapy is fertile ground for psychotherapy integration—acknowledging the limitations of each narrow approach when it comes to explaining and treating complex individuals—and for theoretical pluralism (Caspar, 2008, p. 77; Caspar & Grawe, 1989). In Germany, beginning some 35 years ago, when training was still less formalized, there has been a trend among employers to prefer therapists who have had at least partial training in more than one orientation (e.g., behavior therapy plus gestalt, or plus some psychodynamic elements). Since training has been regulated by law, there seems to be more identification with and concentration on one approach, but trainings usually include some elements from other approaches. Formally, however, integration is forbidden in the strongly approach-oriented regulation of psychotherapy practice in Germany (Caspar, 2008).

In reality, the process of integration begins early: In a poll of 78 trainees undertaken for Caspar (2010) in a convenience sample of participants in postgraduate CBT training in Switzerland and Germany, a majority reported that their supervisors also proposed non-CBT-*concepts*, and even more frequently that they proposed non-CBT *interventions*. "When the supervisees brought in such concepts/interventions they felt strongly supported by their supervisors. The trainees reported furthermore that the inclusion of non-CBT elements was useful for the individual therapies, and they reported with overwhelming clarity that this inclusion increased their therapeutic expertise" (Caspar, 2010, p. 18).

## The Crucial Role of Supervisors

In such an opening up, supervisors are of crucial importance, and their tasks are manifold: To help the trainees recognize their development, to encourage and guide the search for appropriate concepts and procedures, to give support in tolerating ambiguity and complexity, to give feedback and guidance with case formulations, to help with procedures the therapist had not originally learned, including role playing with the therapist to teach a technique, to stabilize the therapist when s/he becomes temporarily desperate, but also to challenge when a supervisee avoids relevant interventions due to personal anxieties.

Halgin (1985) has formulated that

“supervisors play a critical role in escorting beginners through their experiences of artificial security, subsequent confusion, and onward to a process of integration. The supervisor who pushes a beginner into an inappropriate affiliation with a singular model is really colluding with the beginner’s simplistic notion that there might indeed be only one correct way of doing therapy. Such a supervisor is not likely to be sensitive to the struggles of the beginner who is trying to make sense of an overwhelming number of theories and techniques. This beginning period in an individual’s professional development provides an excellent opportunity for communicating the importance of developing integrated methodologies, for it is during this period that the individual is most malleable” (p. 560; Caspar, 2010, pp. 19-20).

If one assumes

"that every patient requires a unique combination of concepts and interventions to best fit and treat the case, things become more complex (FC: than in a narrowly manualized procedure)—and possibly more integrative. This has implications for the supervisor’s tasks: S/he also needs to supervise the selection and use of these concepts and interventions. Castonguay (2000) recommends that a deliberate decision be made as to whether a supervisee wishes to stay within a single therapeutic approach, or to take an integrative perspective. If supervisee and supervisor decide on an integrative stance, concepts and interventions may be chosen from a wide menu." (Caspar, 2010, p. 19).

If the decision is in favor of "a wide menu", the supervisor's task varies: It "depends on the therapist: It may be to convey concepts a less knowledgeable therapist is unfamiliar with, or it may be to help a therapist overwhelmed by the range of possibilities to sort out, decide, and manage complexity in order to maintain the capacity to act." (Caspar, 2010, p. 19).

A good supervisor helps a therapist take the issue of fit between therapist and procedure seriously, and then to deliberate. This is not a trivial task, as it may be difficult to decide whether the view and procedure a therapist decides on is completely appropriate and in the interest of a patient, or whether the therapist imposes his or her own preferences on a patient at the disadvantage of the latter." The individualization of the psychotherapy training process has long been proposed (Caspar, 1997), but has for a variety of reasons made little progress overall. The extent to which therapists should become integrative as opposed to limiting themselves in the scope of concepts considered is certainly an aspect which can and should be considered explicitly independent of other aspects of individualization: "In any case, the supervisor should also reflect with the therapist on the extent to which the use of an integrative stance is actually advantageous in comparison with a pure approach. In spite of a general preference for flexibility expressed here, it is important that before an integrative approach is chosen, it must be better for each patient and situation." (Caspar, 2010, p. 19).

## When and How

The attitude related to *when and how* trainees should be confronted with a transtheoretical stance is clear: One should not spare them from the view that as experience grows, they are expected to gain in transtheoretical knowledge and skills in handling and using this knowledge. Yet the point of departure remains an integrative, but coherent approach (Psychological Therapy, Grawe, 2004), it is communicated that they are not expected to master unlimited complexity, that it is okay to initially be guided by simple rules which give them security and comfort – absolutely essential when they do their first steps as therapists!

Excellent pre-post effect sizes for novice therapists with patients with a broad range of diagnoses, comorbidity and severity at our outpatient clinic at the University of Bern (grosse Holtforth et al., 2011) illustrate that such a practice is feasible and does not lead to overburdening, although novices certainly struggle with complexity.

Now back to the question "What kind of treatment and what kind of therapist would you look for?" Whatever further details are considered: We truly hope that you would go for a therapist open to a transtheoretical stance and educated in several approaches, or in useful conceptual and technical *elements* of such approaches.
